# Uncovering the Molecular Pathways Implicated in the Anti-Cancer Activity of the Imidazoquinoxaline Derivative EAPB02303 Using a *Caenorhabditis elegans* Model

**DOI:** 10.3390/ijms25147785

**Published:** 2024-07-16

**Authors:** Perla Makhoul, Simon Galas, Stéphanie Paniagua-Gayraud, Carine Deleuze-Masquefa, Hiba El Hajj, Pierre-Antoine Bonnet, Myriam Richaud

**Affiliations:** 1 Institut des Biomolécules Max Mousseron (IBMM), UMR 5247, CNRS, ENSCM, Université de Montpellier, 34090 Montpellier, France; perla.makhoul@etu.umontpellier.fr (P.M.); simon.galas@umontpellier.fr (S.G.); stephanie.paniagua-gayraud@umontpellier.fr (S.P.-G.); carine.masquefa@umontpellier.fr (C.D.-M.); 2 Department of Biology, Faculty of Sciences, GSBT Laboratory, Lebanese University, R. Hariri Campus, Hadath 1533, Lebanon; 3 Department of Experimental Pathology, Immunology and Microbiology, Faculty of Medicine, American University of Beirut, Riad El-Solh, P.O. Box 11-0236, Beirut 1107, Lebanon; he21@aub.edu.lb

**Keywords:** *Caenorhabditis elegans*, imiqualines, cancer, IIS pathway, DAF-16/FOXO, PI3K-Akt, Ras-MAPK pathway, LET-60/Ras

## Abstract

Imiqualines are analogues of the immunomodulatory drug imiquimod. EAPB02303, the lead of the second-generation imiqualines, is characterized by significant anti-tumor effects with IC50s in the nanomolar range. We used *Caenorhabditis elegans* transgenic and mutant strains of two key signaling pathways (PI3K-Akt and Ras-MAPK) disrupted in human cancers to investigate the mode of action of EAPB02303. The ability of this imiqualine to inhibit the insulin/IGF1 signaling (IIS) pathway via the PI3K-Akt kinase cascade was explored through assessing the lifespan of wild-type worms. Micromolar doses of EAPB02303 significantly enhanced longevity of N2 strain and led to the nuclear translocation and subsequent activation of transcription factor DAF-16, the only forkhead box transcription factor class O (Fox O) homolog in *C. elegans*. Moreover, EAPB02303 significantly reduced the multivulva phenotype in *let-60*/Ras mutant strains MT2124 and MT4698, indicative of its mode of action through the Ras pathway. In summary, we showed that EAPB02303 potently reduced the activity of IIS and Ras-MAPK signaling in *C. elegans*. Our results revealed the mechanism of action of EAPB02303 against human cancers associated with hyperactivated IIS pathway and oncogenic Ras mutations.

## 1. Introduction

Imiquimod is an immunomodulatory agent [1] marketed as a cream (ALDARA^®^) and approved for the treatment of certain types of skin cancer [2,3] and external genital warts [4]. A family of imiquimod analogues with low molecular weight called imidazoquinoxalines, or imiqualines, was synthesized [5,6]. Two hits from first-generation imiqualines, EAPB0203 and EAPB0503, displayed a pronounced anti-cancer activity at micromolar doses against melanoma [7], acute and chronic myeloid leukemia [8,9,10], and adult T cell leukemia/lymphoma [11,12]. EAPB02303, the lead of the second-generation molecules, showed an enhanced and more potent anti-proliferative effect with IC50s in the nanomolar range, on a panel of cancer cell lines in vitro. While the two most active compounds from the first generation showed an inhibition of tubulin polymerization, transcriptomic analysis of EAPB02303 confirmed a different mechanism of action that remains to be identified [13].

The nematode *Caenorhabditis elegans* is extensively used as a model system for the study of complex molecular processes involved in tumorigenesis [14,15,16]. This model presents numerous advantages that render it an enticing small organism for cancer research [15]. In fact, biological processes involved in mammalian cell proliferation and cell signaling are conserved in the worm [17]. Additionally, at least 83% of the *C. elegans* proteome has human homologs [18], and 72% of tumor driver genes in humans have one or more orthologs in *C. elegans* [16]. Moreover, mutations in key signaling cascades that can promote cancer in humans (namely, PI3K-Akt and Ras-MAPK, among others) are also found in *C. elegans*, and are associated with well-established phenotypes such as longevity and vulva-less or multivulva formation, respectively [19]. Therefore, *C. elegans* is a highly amenable multicellular organism for the in vivo identification of therapeutic targets of anti-tumor agents.

In the present study, we used *C. elegans* as a screening model system to unravel signaling cascades underlying its potent anti-cancer activity. The insulin/insulin-like growth factor 1 (IGF-1) signaling (IIS) pathway is constitutively active in a large number of human solid tumors [20,21]. This pathway regulates *C. elegans* lifespan through the activation of the PI3K-Akt kinase cascade [22], followed by inhibitory phosphorylation of the transcription factor DAF-16 [22,23]. DAF-16 represents the sole ortholog of mammalian forkhead box transcription factors class O (FoxO) [24,25]. Positive modulation of *C. elegans* longevity can be achieved as a result of environmental stressors such as a reduction in bacterial food intake [26,27], but also by direct downregulation of the IIS pathway [28]. Consequently, unphosphorylated DAF-16/FOXO translocates into the nucleus and then promotes the expression of longevity-promoting genes, like *sod-3* (the superoxide dismutase 3), among others [29,30]. Evidently, mutations that decrease IIS pathway activity extend the lifespan in the worm, and alternatively, increase tumor resistance in mammals [31,32]. On the other hand, mutations leading to over-activation of the Ras-MAPK pathway have been frequently reported in melanoma, hairy cell leukemia, non-small cell lung cancer, and thyroid, ovarian, and colorectal cancers [33]. The Ras-MAPK pathway has orthologous components in the worm where it regulates the formation of the vulva [34]. Notably, mutations that result in an enhanced activity of LET-60/Ras in *C. elegans* are analogous to those observed in human cancers [34,35,36,37]. *Let-60*/Ras gain in function mutation leads to an abnormal proliferation of the vulval tissues in adult mutants and the formation of vulval-like protrusions called “pseudovulvas” or “ectopic vulvas”, thus resulting in the multivulva (Muv) phenotype [38]. Previous studies demonstrated that drugs capable of reversing Ras mutant phenotypes in *C. elegans* have the potential to inhibit tumor growth in humans [38,39,40,41]. 

Herein, we verified if the IIS and Ras-MAPK molecular pathways are involved in the anti-tumor activity of EAPB02303. First, we conducted survival analysis using N2 wild-type strain to investigate the effect of varying concentrations of EAPB02303 on IIS pathway regulating *C. elegans* longevity. This notion was further supported by the subsequent analysis of the cellular localization of transcription factor DAF-16/FOXO following treatment with micromolar doses of EAPB02303 using transgenic strain TJ356 expressing the DAF-16::GFP fusion protein. Ultimately, SOD-3 activity was analyzed in the transgenic strain CF1553 harboring SOD-3::GFP after treatment with the molecule EAPB02303. Finally, we used mutant strains harboring a *let-60*/Ras hyperactivation mutation (MT2124 *let-60(n1046gf)* and MT4698 *let-60(n1700gf)*) to study the impact of EAPB02303 on excessive Ras signaling by scoring the Muv phenotype post-treatment. Taken all together, our results revealed for the first time the mechanism of action of EAPB02303 in an in vivo model and showed that its anti-cancer activity is mediated by interfering with both the IIS and Ras-MAPK molecular pathways.

## 2. Results

### 2.1. Micromolar Doses of EAPB02303 Significantly Increase the Lifespan of N2 Wild-Type C. elegans Strain

**Figure 1:**
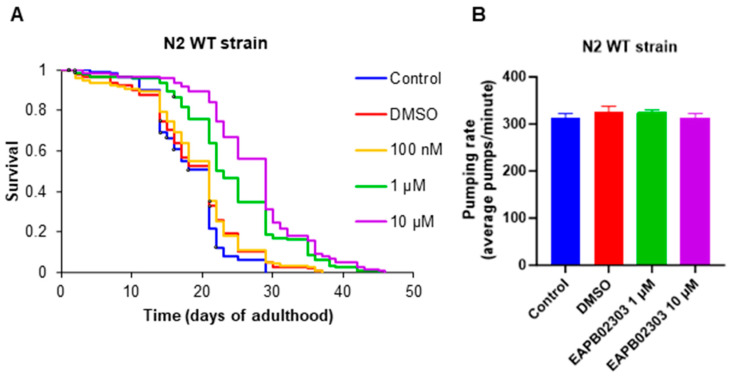
EAPB02303 increases lifespan of N2 wild-type worms without affecting feeding behavior. (**A**) Survival curve of N2 adults. Results represent the average of two independent experiments (n = 120). (**B**) Average pumping rate of N2 young adults. Results shown represent the average of three independent experiments with ±SD (n = 30). One-way ANOVA was performed to validate significance. Results are non-significant compared to control.

*C. elegans* lifespan can be subject to disruption by treatments influencing signal transduction cascades that regulate longevity in the worm, particularly the IIS pathway [42]. While the 100 nM dose had no effect on the longevity of the N2 wild-type strain, worms treated with either 1 µM or 10 µM EAPB02303 displayed a highly significant lifespan extension compared to the control and vehicle DMSO groups (*p*-value < 0.0001) (Figure 1A, Table 1). This suggests that EAPB02303 causes a reduction in IIS pathway activity.

### 2.2. EAPB02303 Does Not Affect N2 Wild-Type Strain Pharyngeal Pumping Rates

To investigate whether the worms’ lifespan extension following treatment with EAPB02303 is not the result of a decrease in feeding behavior, pharyngeal pumping rates were measured as previously described [43]. The average pumping activity remained unchanged in treated groups, as compared to the control untreated group (Figure 1B), suggesting that the observed increase in *C. elegans* lifespan is not induced by dietary restriction, but it rather results from a modulation of the IIS pathway mediated by EAPB02303. 

### 2.3. EAPB02303 Leads to the Nuclear Translocation and Activation of DAF-16/FOXO and Prolongs Survival of C. elegans through DAF-16 Independently from Sod-3

**Figure 2:**
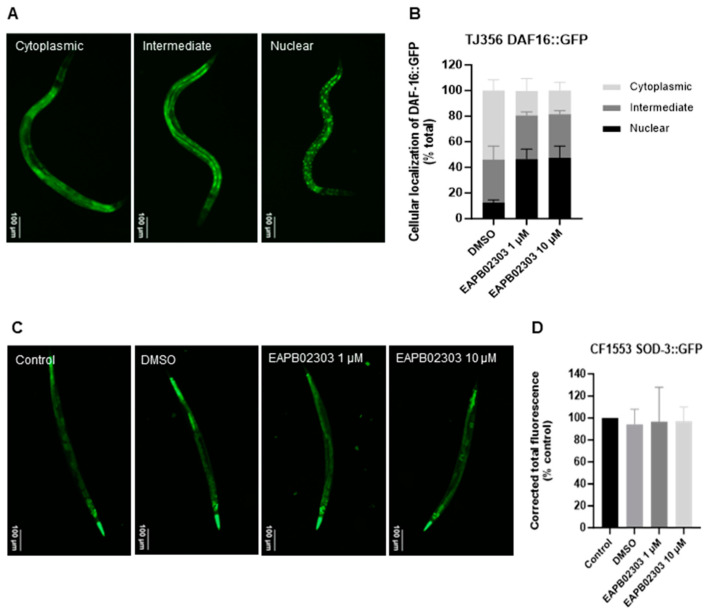
EAPB02303 induces DAF-16::GFP nuclear translocation without activating SOD-3. (**A**) Representative images of DAF-16::GFP in *C. elegans* transgenic strain TJ356. (**B**) Quantification of DAF-16::GFP cellular localization in TJ356 worms. Cellular localization of DAF-16 was determined using fluorescent microscopy as follows: cytoplasmic, intermediate, or nuclear. Results shown represent the average of two independent experiments with ±SD (n = 200). Two-way ANOVA was performed to validate significance of DAF-16 cellular localization post-treatment compared to the respective DMSO group presenting the same localization as follows: nuclear (DMSO vs. 1 µM or 10 µM), intermediate (DMSO vs. 1 µM or 10 µM: non-significant), and cytoplasmic (DMSO vs. 1 µM or 10 µM). (**C**) Representative images of SOD-3::GFP expression in *C. elegans* transgenic strain CF1553. (**D**) Fluorescence quantification of CF1553 worms harboring SOD-3::GFP. Results shown represent the average of five independent experiments with ±SD (n = 100). One-way ANOVA was performed to validate significance. Results are non-significant compared to control.

The transcription factor DAF-16/FOXO activity status was assessed to unveil how EAPB02303 prolongs worm longevity. While phosphorylated inactive DAF-16/FOXO is retained in the cytoplasm, an intermediate or nuclear localization of DAF-16/FOXO indicates a corresponding intermediate or full activation status (Figure 2A). Using transgenic strain TJ356 expressing the DAF-16::GFP fusion protein, a drastic increase was noted in nuclear fluorescence from 12% under control conditions to 47% after three-day exposure of L4 synchronized worms to 1 µM or 10 µM EAPB02303 (*p*-value < 0.01) (Figure 2B), reflecting the activation and subsequent nuclear translocation of DAF-16/FOXO upon treatment with this compound.

We then analyzed the activation of *sod-3* using the transgenic strain CF1553 harboring SOD-3::GFP, following a three-day treatment of synchronized L4 worms with micromolar doses of EAPB02303 (Figure 2C). EAPB02303 did not change the expression of SOD-3 (Figure 2D), indicating that EAPB02303 lifespan extension is mediated through DAF-16, which acts independently from SOD-3.

### 2.4. EAPB02303 Reduces the Multivulva Phenotype in Let-60 Mutants

**Figure 3:**
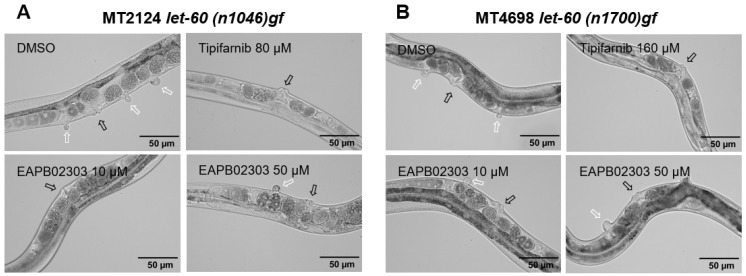

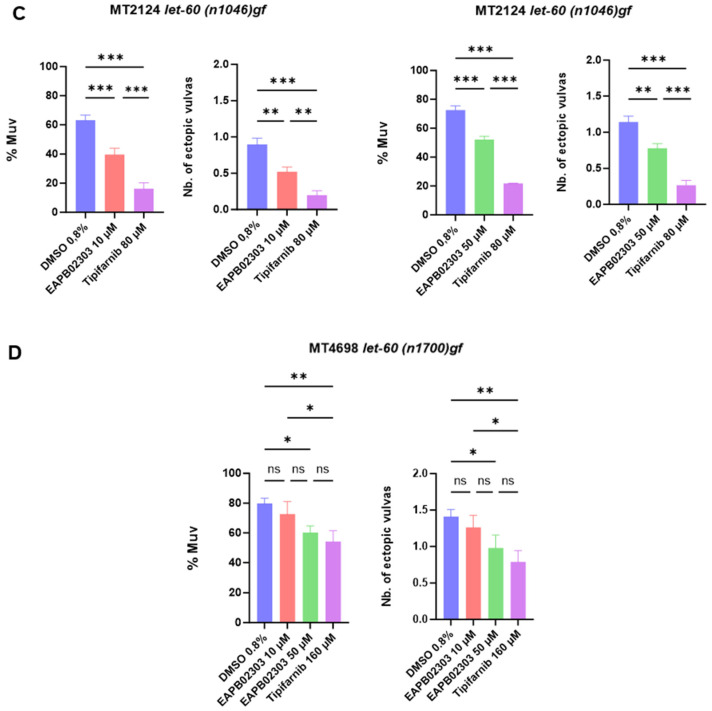
EAPB02303 significantly reduces the Muv phenotype caused by *let-60*/Ras hyperactivation mutation in MT2124 and MT4698 mutant strains. (**A**) Representative images of Muv phenotype in MT2124 *let-60(n1046)gf* and in (**B**) MT4698 *let-60(n1700)gf* mutant strains. White arrows indicate protruding ectopic vulvas, black arrows indicate normal vulvas. (**C**) Number of worms from the mutant strain MT2124 *let-60(n1046)gf* expressing the Muv phenotypic feature and the number of ectopic vulvas per nematode were scored. Results shown represent the average of three independent replicates ± SD (n = 400). (**D**) Number of worms from the mutant strain MT4698 *let-60(n1700)gf* expressing the Muv phenotypic feature and the number of ectopic vulvas per animal were scored. Results shown represent the average of 3 independent replicates ± SD (n = 300). One-way ANOVA was performed to validate significance as compared to control as follows: * *p*-value < 0.05, ** *p*-value < 0.01, and *** *p*-value < 0.001.

To identify other potential molecular pathways implicated in activity of EAPB02303, we used *let-60*/Ras mutant strains (MT2124 *let-60(n1046gf)* and MT4698 *let-60(n1700gf)*) carrying a missense mutation at codon 13 (G13E) in alleles *n1064* and *n1700*, respectively (Figure 3A,B) [38]. Tipifarnib, a Ras farnesyltransferase inhibitor, was used as a positive control as previously described [38]. In MT2124 *let-60(n1046gf)*, 10 µM EAPB02303 significantly reduced the number of Muv individuals from 63% (DMSO group) to 40% after treatment, in addition to a potent decrease in the number of pseudovulvas per animal (*p*-value < 0.001) (Figure 3C). Similar results were obtained using higher concentration of EAPB02303 (50 µM), whilst 80 µM tipifarnib almost completely suppressed the Muv phenotype (Figure 3C).

In MT4698 *let-60(n1700gf)*, EAPB02303 at 10 µM displayed no Muv-suppressive properties and the Muv population remained the same compared to the DMSO cohort (Figure 3D). In contrast, a higher dose of 50 µM induced significant decrease in the number of Muv animals to less than 60% (*p*-value < 0.05), and a significant reduction in the frequency of ectopic vulvas per worm was also observed (*p*-value < 0.05) (Figure 3D). These results imply that EAPB02303 reverses the Muv phenotype caused by the *let-60*/Ras over-expression in both strains and not the genetic background of MT2124 *let-60(n1046)gf*. 

## 3. Discussion

*C. elegans* has been gaining growing interest as a model organism for research on cancer. In fact, up to 72% of cancer genes are conserved from humans to the nematode [16], and 83% of the *C. elegans* proteome has human homologs [18]. Although mutations in well-known signaling cascades that can promote cancer in humans are conserved in *C. elegans*, they do not result in the formation of malignant tumors in the worm. However, these mutations are associated with distinct, well-established, and noticeable phenotypes. Therefore, the screening for the mechanism of action of novel drugs relies on the study of molecules that could reverse these characteristic features in *C. elegans* mutant strains [39]. Indeed, if worms can be treated with a drug of interest through feeding or direct contact, then these molecules can reach *C. elegans* cells via the intestine or by passive diffusion through the cuticle [44]. Previous studies have used *C. elegans* as a tool to elucidate the mechanism of action of anti-cancer drugs [19,38,39,45,46,47], hence serving as a proof of concept that the signaling pathways involved in the mode of action could be conserved in human tumor cells. In this study, we deciphered the mechanism of action of EAPB02303, a second-generation imiqualine, in *C. elegans*. We showed that EAPB02303 is active at low micromolar concentrations and results in a significant lifespan extension of adult N2 wild-type worms, without impacting their feeding behavior. This means that EAPB02303 is digested by the worms and/or diffuses through their cuticle, resulting in an increased longevity. *C. elegans* lifespan is mainly regulated by the IIS pathway, via an insulin/IGF-like receptor followed by a cascade of downstream kinases (AGE-1 > PDK-1 > AKT-1/2) and a transcription factor (DAF-16) [22], all of which have mammalian counterparts (PI3K > PDK > Akt > FOXO). Our data suggest that EAPB02303 reduces the insulin signaling via PI3K-Akt, a cascade that is frequently aberrantly activated in many human cancers rendering it an attractive druggable target [48]. This observation was subsequently accompanied by DAF-16/FOXO activation and nuclear translocation following treatment with EAPB02303. Our results are in accordance with several studies which demonstrate that a decrease in the IIS followed by DAF-16/FOXO activation results in an increase in *C. elegans* longevity and, alternatively, raises tumor resistance in mammals [31,32]. Indeed, previous findings highlight the role of FOXO transcription factors as tumor suppressors due to their pro-apoptotic and anti-proliferative activities in leukemias [49,50], pancreatic [51], prostate [52,53], breast [54,55], and thymic cancers [56]. Our results presumably delineate that EAPB02303 exerts its anti-cancer action via FOXO activation, which remains to be validated in human cells. Upon reduced IIS activity, DAF-16/FOXO activation and nuclear translocation results in the upregulation and downregulation of a panel of genes, among which is the mitochondrial superoxide dismutase *sod-3* [30,57]. To our surprise, we did not observe any changes in the level of expression of SOD-3 after treatment with EAPB02303. Nonetheless, *sod-3* is but one effector gene of a vast, intricate network downstream of DAF-16/FOXO [30,57]; hence, a myriad of other genes could impact the lifespan as a response to EAPB02303.

On the other hand, the Ras-MAPK pathway (Ras > Raf > Mek > Erk), which controls mammalian cell divisions, is frequently mutated in human cancers [33]. Over-activated Ras protein is a perturbation commonly associated with one-third of human cancers and malignancies [58]. Evidently, Ras mutations substantially contribute to cancer progression and maintenance [59,60]. This pathway has orthologous counterparts in the worm (LET-60 > LIN-45 > MEK-2 > MPK-1), where it regulates vulva formation [34]. Most importantly, mutations that result in an enhanced activity of LET-60/Ras in *C. elegans* are analogous to those observed in human cancers [34,35,36,37]. *Let-60*/Ras gain in function mutation leads to an abnormal proliferation of the vulval tissues in adult mutants resulting in formation of the multivulva (Muv) phenotype [38]. We showed that EAPB02303 significantly reverses the Muv trait caused by *let-60*/Ras hyperactivation mutation at the low dose of 10 µM. Our results were in line with previous findings which highlight that drugs capable of reversing Ras mutant phenotypes in *C. elegans* have the potential to inhibit tumor growth in humans [38,39,40,41]. Foremost, we showed that EAPB02303 is active at a dose that is eight times lower than a clinically used imidazole farnesyltransferase inhibitor tested in *C. elegans* and other natural compounds with Ras-inhibitory activity [38]. Our compound demonstrated remarkable potency against *let-60*/Ras mutants, at a concentration over a million times lower than five candidate drugs that were previously screened in *C. elegans*, with similar anti-Ras activity [39]. Our data outline the Ras-inhibitory effect of EAPB02303 and crucially, the remarkable anti-cancer potency of this molecule as compared to other drugs.

**Figure 4:**
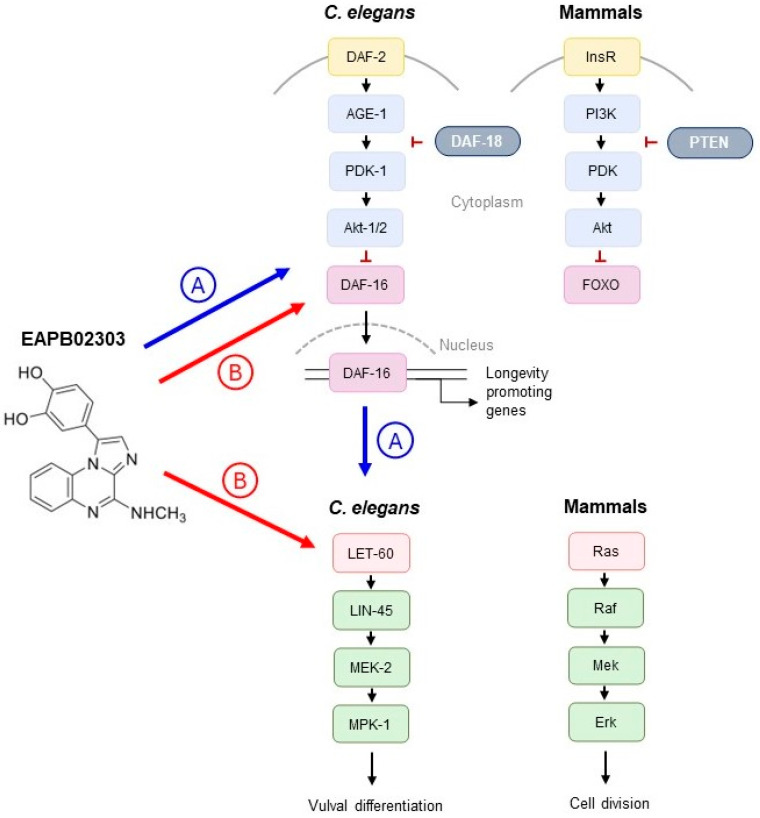
Representation of the IIS pathway controlling worm longevity (adapted from [65,66]), and the Ras-MAPK pathway, which determines vulval differentiation (adapted from [38]) compared to their respective mammalian counterparts regulating cellular divisions and the proposed mechanism of action of EAPB02303. (A) EAPB02303 decreases IIS activity first, followed by consequent decrease in Ras-MAPK pathway activity resulting from the interconnectivity between IIS/Ras-MAPK cascades [63]. (B) EAPB02303 results in simultaneous reduction in signaling activity of IIS and Ras-MAPK pathways.

At the molecular level, the IIS and Ras-MAPK signaling pathways exhibit a complex interconnectivity that is conserved across species [61]. Prior investigations showed that there is indeed cross-talk between the IIS and Ras-MAPK signaling cascades in *C. elegans* [62,63,64]. Some studies demonstrated that Ras signaling promotes longevity without influencing DAF-16/FOXO cellular localization [62]. By contrast, we observed that EAPB02303 increases longevity via nuclear translocation of DAF-16/FOXO and significantly reduces Ras-MAPK signaling activity. However, contrary to the former study [62], others showed that reduced DAF-2 activity suppresses the Muv phenotype in *let-60(gf)* mutants [63,64]. The mechanism of action of our compound could therefore be supported by the latter. This implies that initially, EAPB02303 reduces the IIS pathway activity, and subsequently, the decrease in IIS reverses the Muv phenotype caused by hyperactivated *let-60*/Ras (Figure 4A). However, this does not eliminate the prospect of simultaneous reduction in signaling activity of both pathways following treatment with EAPB02303 (Figure 4B). These two possibilities regarding the precise mode of action of EAPB02303 can be confirmed through future research.

In conclusion, our results unveil the anti-cancer mechanism of action of the second-generation imiqualine EAPB02303 in *C. elegans.* EAPB02303 displayed IIS- and Ras-inhibitory activity, thus holding promising therapeutic expectations in malignant tumors with deregulated PI3K-Akt signaling and/or oncogenic Ras. In the future, we are intrigued by the prospect of substantiating our findings through validation in human cancer cell lines associated with hyperactivated IIS or Ras activity, such as cutaneous melanoma and certain types of leukemias, among many other human malignancies [20,21,33]. Nevertheless, we were able to demonstrate promising molecular pathways implicated in the mode of action of EAPB02303 for the first time using a simple in vivo model such as *C. elegans*.

## 4. Materials and Methods

### 4.1. C. elegans Strains and Maintenance

The following strains were obtained from the Caenorhabditis Genetics Center (CGC) at the University of Minnesota, which is funded by NIH Office of Research Infrastructure Programs (P40 OD010440): wild type Bristol (N2), TJ356 (zIs356 IV. [daf-16p::daf-16a/b::GFP + rol-6(su1006)]), CF1553 (muIs84 [(pAD76) sod-3p::GFP + rol-6(su1006)]), MQ887 (isp-1(qm150) IV.), MQ130 (clk-1(qm30) III.), MT2124 (*let-60(n1046)* IV.), and MT4698 (*let-60(n1700)* IV.). Worms were maintained at 20 °C (mitochondrial mutants were maintained at 15 °C as recommended) on Nematode Growth Medium (NGM) Petri dishes with *Escherichia coli* HT115(DE3) as food source (CGC). 

### 4.2. C. elegans Synchronization

Worms were cultured and monitored until the maximal egg laying was reached. Plates were washed using M9 buffer and the surface of the NGM agar was gently scraped to collect all worms and eggs. Next, eggs were treated with the bleaching solution (NaOH 5M and household bleach) for 3 min with frequent agitation, then washed with M9 to stop the reaction. The eggs were collected in S-medium and transferred into a new NGM plate seeded with H115(DE3) *Escherichia coli* bacteria. Worms were then selected at the desired stage for the rest of the experiments.

### 4.3. Preparation of Drugs

EAP02303 was synthesized by “Oncopharmacochemistry and Cutaneous Pharmacotoxicology” laboratory (Institut des Biomolécules Max Mousseron IBMM, Montpellier University, Montpellier, France) as previously described [13]. EAPB02303 was dissolved in dimethyl sulfoxide (DMSO) to prepare a stock solution of 10^−2^ M, aliquoted and stored at −20 °C. Tipifarnib (Sigma-Aldrich (St. Louis, MO, USA) Cas No 192185-72-1) was dissolved in DMSO to prepare a stock solution of 20 mM, aliquoted and stored at −20 °C. The negative control used was DMSO percentage corresponding to the highest working concentration.

### 4.4. Pharyngeal Pumping Assay

Drugs were mixed with liquid NGM immediately before pouring into petri dishes, and plates were kept at room temperature until the agar solidified. Synchronized eggs from N2 wild type strain were deposited on NGM seeded with equal amounts of H115(DE3) bacteria, and they were maintained at 20 °C for 3 days. The pharyngeal pumping rate of young adults was measured using LEICA M205 FCA microscope at 16.0× magnification. The number of pharynx pumps by counting grinder movements was scored manually using a clicker counter for 20 s every minute for 10 min [43] and mean pumps per minute was calculated. Three independent replicates were performed with a total of n = 30 worms per condition. 

### 4.5. Lifespan Bioassay

Synchronized N2 adults were transferred from NGM plates into ELISA 96-well plates using the worm picking method. Briefly, a 1-inch piece of a platinum wire was mounted into the tip of a Pasture pipet and five adult worms were individually picked and transferred into each well. Adults were suspended in S-medium containing 5-fluoro-2′-deoxyuridine (FUdR), to sterilize the animals and avoid offspring, and they were fed dead H115(DE3) *E. coli* bacteria to exclude the capacity of living bacteria to metabolize the drugs. Worms were examined daily and monitored for survival. Killed HT155(DE3) bacteria were added to the plates weekly to avoid starvation. Animals that did not display spontaneous movement in the liquid solution or did not respond to gentle touch with a sterilized platinum wire were scored as dead. This experiment was conducted twice with a total of n = 120 worms per group.

### 4.6. DAF-16::GFP Cellular Localization 

L4 synchronized worms from the transgenic strain TJ356 expressing the DAF-16::GFP fusion protein were transferred to 15 mL falcon tubes containing S-medium, FUdR to prevent offspring formation, and inactive HT115(DE3) as food source. Animals were incubated with 1 or 10 µM EAPB02303 at 20 °C for three days. Sub-cellular localization of the transcription factor DAF-16 was determined for each individual using fluorescent microscopy (LEICA M205 FCA) as follows: “cellular”, “intermediate”, or “nuclear” (n = 200 worms per condition).

### 4.7. Quantification of SOD-3::GFP Expression

L4 synchronized worms from the transgenic strain CF1553 expression the superoxide dismutase SOD-3::GFP fusion protein were transferred to 15 mL falcon tubes containing S-medium, FUdR to prevent offspring formation, and inactive HT115(DE3) as food source. Animals were incubated with 1 or 10 µM EAPB02303 at 20 °C for three days. Worms were then transferred to slides and images of each individual were taken at 6.8× magnification using fluorescent microscopy (LEICA M205 FCA). Fluorescence was quantified using ImageJ software version 1.53e and the percentage of corrected total fluorescence was calculated based on the following equation:

Corrected total fluorescence = Integrated density − (Area of selected worm × Mean fluorescence of background readings)

Five biological replicates were performed with a total of n = 100 worms per condition.

### 4.8. Analysis of the Multivulva Phenotype

Phenotypic analysis of the multivulva (Muv) phenotype was performed based on a modified protocol described by Ji et al. [38]. Drugs were mixed with liquid NGM immediately before pouring into petri dishes, and plates were kept at room temperature until the agar solidified. Synchronized eggs from *let-60* mutant strains were deposited on NGM seeded with inactive HT115(DE3) and were maintained at 20 °C for 3 days. Day 1 adults were collected with M9 buffer and mounted on slides to score the percent of Muv phenotype and the number of ectopic vulvas per worm. Adults with one or more protrusions in addition to a normal vulva were classified as Muv. Experiments were carried out in triplicate with a total of n = 300 to 400 worms per group. Images were taken using a Leica DM 6B upright microscope at 20× magnification. 

### 4.9. Statistical Analysis

Kaplan–Meier analysis for the survival data was performed using XLSTAT software version 2022.2.1 1293 (Addinsoft, New York, NY, USA). 

The rest of the data were processed using GraphPad Prism version 10.1.2. The data from the treated groups were compared to the control groups using one-way or two-way ANOVA. The results are presented as the mean ± standard deviation, and the statistical significance was considered as follows: * *p*-value < 0.05, ** *p*-value < 0.01 and *** *p*-value < 0.001.

## Figures and Tables

**Table 1:** Kaplan–Meier analysis of N2 strain survival data.

Condition	Total Observed	Total Censored	Mean Survival Time (Days)	Max Lifespan (Days)	Code	Statistics *
Control	120	12	18.3	29	A	*p* = 0.071 (B) *p* = 0.029 (C) *p* < 0.0001 (D) *p* < 0.0001 (E)
DMSO	120	4	18.9	37	B	*p* = 0.071 (A) *p* = 0.728 (C) *p* < 0.0001 (D) *p* < 0.0001 (E)
100 nM	120	2	19.2	37	C	*p* = 0.029 (A) *p* = 0.728 (B) *p* < 0.0001 (D) *p* < 0.0001 (E)
1 µM	120	2	24.2	45	D	*p* < 0.0001 (A) *p* < 0.0001 (B) *p* < 0.0001 (C) *p* = 0.004 (E)
10 µM	122	1	27.3	46	E	*p* < 0.0001 (A) *p* < 0.0001 (B) *p* < 0.0001 (C) *p* = 0.004 (D)

* Kaplan–Meier analysis performed to display statistical significance after comparisons between the following groups: (A) control, (B) DMSO, (C) 100 nM EAPB02303, (D) 1 µM EAPB02303, and (E) 10 µM EAPB02303.

## Data Availability

The original contributions presented in this study are included in the article; further inquiries can be directed to the corresponding authors.

## References

[1] Rudy S.J. (2002). Imiquimod (Aldara): Modifying the immune response. Dermatol. Nurs..

[2] Steinmann A., Funk J.O., Schuler G., von den Driesch P. (2000). Topical imiquimod treatment of a cutaneous melanoma metastasis. J. Am. Acad. Dermatol..

[3] Bubna A.K. (2015). Imiquimod—Its role in the treatment of cutaneous malignancies. Indian J. Pharmacol..

[4] Hengge U.R., Cusini M. (2003). Topical immunomodulators for the treatment of external genital warts, cutaneous warts and molluscum contagiosum. Br. J. Dermatol..

[5] Cuq P., Deuleuze-Masquefa C., Bonnet P.-A., Patinote C. (2015). New Imidazo[1,2-a]quinoxalines and derivatives for the treatment of cancers. Patent.

[6] Deuleuze-Masquefa C., Moarbess G., Bonnet P.-A., Pinguet F., Bazarbachi A., Bressole F. (2008). Imidazo[1,2-a]quinoxalines and derivatives for the treatment of cancers. Patent.

[7] Deleuze-Masquefa C., Moarbess G., Khier S., David N., Gayraud-Paniagua S., Bressolle F., Pinguet F., Bonnet P.-A. (2009). New imidazo[1,2-a]quinoxaline derivatives: Synthesis and in vitro activity against human melanoma. Eur. J. Med. Chem..

[8] Nabbouh A.I., Hleihel R.S., Saliba J.L., Karam M.M., Hamie M.H., Wu H.J.M., Berthier C.P., Tawil N.M., Bonnet P.A., Deleuze-Masquefa C. (2017). Imidazoquinoxaline derivative EAPB0503: A promising drug targeting mutant nucleophosmin 1 in acute myeloid leukemia. Cancer.

[9] Saliba J., Deleuze-Masquéfa C., Iskandarani A., El Eit R., Hmadi R., Mahon F.X., Bazarbachi A., Bonnet P.A., Nasr R. (2014). EAPB0503, a novel imidazoquinoxaline derivative, inhibits growth and induces apoptosis in chronic myeloid leukemia cells. Anticancer Drugs.

[10] Skayneh H., Jishi B., Hleihel R., Hamie M., El Hajj R., Deleuze-Masquefa C., Bonnet P.A., El Sabban M., El Hajj H. (2022). EAPB0503, an Imidazoquinoxaline Derivative Modulates SENP3/ARF Mediated SUMOylation, and Induces NPM1c Degradation in NPM1 Mutant AML. Int. J. Mol. Sci..

[11] Moarbess G., El-Hajj H., Kfoury Y., El-Sabban M.E., Lepelletier Y., Hermine O., Deleuze-Masquéfa C., Bonnet P.-A., Bazarbachi A. (2008). EAPB0203, a member of the imidazoquinoxaline family, inhibits growth and induces caspase-dependent apoptosis in T-cell lymphomas and HTLV-I–associated adult T-cell leukemia/lymphoma. Blood.

[12] Moarbess G., Deleuze-Masquefa C., Bonnard V., Gayraud-Paniagua S., Vidal J.R., Bressolle F., Pinguet F., Bonnet P.A. (2008). In vitro and in vivo anti-tumoral activities of imidazo[1,2-a]quinoxaline, imidazo[1,5-a]quinoxaline, and pyrazolo[1,5-a]quinoxaline derivatives. Bioorg. Med. Chem..

[13] Patinote C., Deleuze-Masquéfa C., Kaddour K.H., Vincent L.A., Larive R., Zghaib Z., Guichou J.F., Assaf M.D., Cuq P., Bonnet P.A. (2021). Imidazo[1,2-a]quinoxalines for melanoma treatment with original mechanism of action. Eur. J. Med. Chem..

[14] Kirienko N.V., Mani K., Fay D.S. (2010). Cancer models in *Caenorhabditis elegans*. Dev. Dyn..

[15] Kyriakakis E., Markaki M., Tavernarakis N. (2015). *Caenorhabditis elegans* as a model for cancer research. Mol. Cell. Oncol..

[16] Cerón J. (2023). *Caenorhabditis elegans* for research on cancer hallmarks. Dis. Model. Mech..

[17] Harris T.W., Chen N., Cunningham F., Tello-Ruiz M., Antoshechkin I., Bastiani C., Bieri T., Blasiar D., Bradnam K., Chan J. (2004). WormBase: A multi-species resource for nematode biology and genomics. Nucleic Acids Res..

[18] Lai C.H., Chou C.Y., Ch’ang L.Y., Liu C.S., Lin W. (2000). Identification of novel human genes evolutionarily conserved in *Caenorhabditis elegans* by comparative proteomics. Genome Res..

[19] Kobet R.A., Pan X., Zhang B., Pak S.C., Asch A.S., Lee M.H. (2014). *Caenorhabditis elegans*: A Model System for Anti-Cancer Drug Discovery and Therapeutic Target Identification. Biomol. Ther..

[20] Rascio F., Spadaccino F., Rocchetti M.T., Castellano G., Stallone G., Netti G.S., Ranieri E. (2021). The Pathogenic Role of PI3K/AKT Pathway in Cancer Onset and Drug Resistance: An Updated Review. Cancers.

[21] Hua H., Zhang H., Chen J., Wang J., Liu J., Jiang Y. (2021). Targeting Akt in cancer for precision therapy. J. Hematol. Oncol..

[22] Murphy C.T., Hu P.J. (2013). Insulin/insulin-like growth factor signaling in *C. elegans*. WormBook: The Online Review of C. elegans Biology.

[23] Henderson S.T., Johnson T.E. (2001). daf-16 integrates developmental and environmental inputs to mediate aging in the nematode *Caenorhabditis elegans*. Curr. Biol..

[24] Lin K., Dorman J.B., Rodan A., Kenyon C. (1997). daf-16: An HNF-3/forkhead family member that can function to double the life-span of *Caenorhabditis elegans*. Science.

[25] Ogg S., Paradis S., Gottlieb S., Patterson G.I., Lee L., Tissenbaum H.A., Ruvkun G. (1997). The Fork head transcription factor DAF-16 transduces insulin-like metabolic and longevity signals in *C. elegans*. Nature.

[26] Houthoofd K., Vanfleteren J.R. (2006). The longevity effect of dietary restriction in *Caenorhabditis elegans*. Exp. Gerontol..

[27] Kaeberlein T.L., Smith E.D., Tsuchiya M., Welton K.L., Thomas J.H., Fields S., Kennedy B.K., Kaeberlein M. (2006). Lifespan extension in *Caenorhabditis elegans* by complete removal of food. Aging Cell.

[28] Carretero M., Gomez-Amaro R.L., Petrascheck M. (2015). Pharmacological classes that extend lifespan of *Caenorhabditis elegans*. Front. Genet..

[29] Tepper R.G., Ashraf J., Kaletsky R., Kleemann G., Murphy C.T., Bussemaker H.J. (2013). PQM-1 complements DAF-16 as a key transcriptional regulator of DAF-2-mediated development and longevity. Cell.

[30] Murphy C.T., McCarroll S.A., Bargmann C.I., Fraser A., Kamath R.S., Ahringer J., Li H., Kenyon C. (2003). Genes that act downstream of DAF-16 to influence the lifespan of *Caenorhabditis elegans*. Nature.

[31] Pinkston J.M., Garigan D., Hansen M., Kenyon C. (2006). Mutations that increase the life span of *C. elegans* inhibit tumor growth. Science.

[32] Pinkston-Gosse J., Kenyon C. (2007). DAF-16/FOXO targets genes that regulate tumor growth in *Caenorhabditis elegans*. Nat. Genet..

[33] Burotto M., Chiou V.L., Lee J.M., Kohn E.C. (2014). The MAPK pathway across different malignancies: A new perspective. Cancer.

[34] Beitel G.J., Clark S.G., Horvitz H.R. (1990). *Caenorhabditis elegans* ras gene let-60 acts as a switch in the pathway of vulval induction. Nature.

[35] Ferguson E.L., Horvitz H.R. (1985). Identification and characterization of 22 genes that affect the vulval cell lineages of the nematode *Caenorhabditis elegans*. Genetics.

[36] Bos J.L. (1989). ras oncogenes in human cancer: A review. Cancer Res..

[37] Han M., Aroian R.V., Sternberg P.W. (1990). The let-60 locus controls the switch between vulval and nonvulval cell fates in *Caenorhabditis elegans*. Genetics.

[38] Ji J., Yuan J., Guo X., Ji R., Quan Q., Ding M., Li X., Liu Y. (2019). Harmine suppresses hyper-activated Ras–MAPK pathway by selectively targeting oncogenic mutated Ras/Raf in *Caenorhabditis elegans*. Cancer Cell Int..

[39] Medina P.M., Ponce J.M., Cruz C.A. (2021). Revealing the anticancer potential of candidate drugs in vivo using *Caenorhabditis elegans* mutant strains. Transl. Oncol..

[40] Liu Y., Zhi D., Li M., Liu D., Wang X., Wu Z., Zhang Z., Fei D., Li Y., Zhu H. (2016). Shengmai Formula suppressed over-activated Ras/MAPK pathway in *C. elegans* by opening mitochondrial permeability transition pore via regulating cyclophilin D. Sci. Rep..

[41] Liu D., Zhi D., Zhou T., Yu Q., Wan F., Bai Y., Li H. (2013). Realgar bioleaching solution is a less toxic arsenic agent in suppressing the Ras/MAPK pathway in *Caenorhabditis elegans*. Environ. Toxicol. Pharmacol..

[42] Kenyon C. (2005). The plasticity of aging: Insights from long-lived mutants. Cell.

[43] Raizen D., Song B.M., Trojanowski N., You Y.J. (2012). Methods for measuring pharyngeal behaviors. WormBook: The Online Review of C. elegans Biology.

[44] Zheng S.Q., Ding A.J., Li G.P., Wu G.S., Luo H.R. (2013). Drug absorption efficiency in Caenorhbditis elegans delivered by different methods. PLoS ONE.

[45] Sánchez-Blanco A., Rodríguez-Matellán A.G., Reis-Sobreiro M., Sáenz-Narciso B., Cabello J., Mohler W.A., Mollinedo F. (2014). *Caenorhabditis elegans* as a platform to study the mechanism of action of synthetic antitumor lipids. Cell Cycle.

[46] Bae Y.K., Sung J.Y., Kim Y.N., Kim S., Hong K.M., Kim H.T., Choi M.S., Kwon J.Y., Shim J. (2012). An in vivo *C. elegans* model system for screening EGFR-inhibiting anti-cancer drugs. PLoS ONE.

[47] Henarejos-Escudero P., Hernández-García S., Guerrero-Rubio M.A., García-Carmona F., Gandía-Herrero F. (2020). Antitumoral Drug Potential of Tryptophan-Betaxanthin and Related Plant Betalains in the *Caenorhabditis elegans* Tumoral Model. Antioxidants.

[48] Farhan M., Wang H., Gaur U., Little P.J., Xu J., Zheng W. (2017). FOXO Signaling Pathways as Therapeutic Targets in Cancer. Int. J. Biol. Sci..

[49] Kikuchi S., Nagai T., Kunitama M., Kirito K., Ozawa K., Komatsu N. (2007). Active FKHRL1 overcomes imatinib resistance in chronic myelogenous leukemia-derived cell lines via the production of tumor necrosis factor-related apoptosis-inducing ligand. Cancer Sci..

[50] Ticchioni M., Essafi M., Jeandel P.Y., Davi F., Cassuto J.P., Deckert M., Bernard A. (2007). Homeostatic chemokines increase survival of B-chronic lymphocytic leukemia cells through inactivation of transcription factor FOXO3a. Oncogene.

[51] Roy S.K., Srivastava R.K., Shankar S. (2010). Inhibition of PI3K/AKT and MAPK/ERK pathways causes activation of FOXO transcription factor, leading to cell cycle arrest and apoptosis in pancreatic cancer. J. Mol. Signal..

[52] Lynch R.L., Konicek B.W., McNulty A.M., Hanna K.R., Lewis J.E., Neubauer B.L., Graff J.R. (2005). The progression of LNCaP human prostate cancer cells to androgen independence involves decreased FOXO3a expression and reduced p27KIP1 promoter transactivation. Mol. Cancer Res..

[53] Li Y., Wang Z., Kong D., Murthy S., Dou Q.P., Sheng S., Reddy G.P., Sarkar F.H. (2007). Regulation of FOXO3a/beta-catenin/GSK-3beta signaling by 3,3′-diindolylmethane contributes to inhibition of cell proliferation and induction of apoptosis in prostate cancer cells. J. Biol. Chem..

[54] Sunters A., Madureira P.A., Pomeranz K.M., Aubert M., Brosens J.J., Cook S.J., Burgering B.M., Coombes R.C., Lam E.W. (2006). Paclitaxel-induced nuclear translocation of FOXO3a in breast cancer cells is mediated by c-Jun NH2-terminal kinase and Akt. Cancer Res..

[55] Eddy S.F., Kane S.E., Sonenshein G.E. (2007). Trastuzumab-resistant HER2-driven breast cancer cells are sensitive to epigallocatechin-3 gallate. Cancer Res..

[56] Paik J.H., Kollipara R., Chu G., Ji H., Xiao Y., Ding Z., Miao L., Tothova Z., Horner J.W., Carrasco D.R. (2007). FoxOs are lineage-restricted redundant tumor suppressors and regulate endothelial cell homeostasis. Cell.

[57] Murphy C.T. (2006). The search for DAF-16/FOXO transcriptional targets: Approaches and discoveries. Exp. Gerontol..

[58] Karnoub A.E., Weinberg R.A. (2008). Ras oncogenes: Split personalities. Nat. Rev. Mol. Cell Biol..

[59] Saito R.M., van den Heuvel S. (2002). Malignant worms: What cancer research can learn from *C. elegans*. Cancer Investig..

[60] Chin L., Tam A., Pomerantz J., Wong M., Holash J., Bardeesy N., Shen Q., O’Hagan R., Pantginis J., Zhou H. (1999). Essential role for oncogenic Ras in tumour maintenance. Nature.

[61] Laskovs M., Partridge L., Slack C. (2022). Molecular inhibition of RAS signalling to target ageing and age-related health. Dis. Models Mech..

[62] Nanji M., Hopper N.A., Gems D. (2005). LET-60 RAS modulates effects of insulin/IGF-1 signaling on development and aging in *Caenorhabditis elegans*. Aging Cell.

[63] Battu G., Hoier E.F., Hajnal A. (2003). The *C. elegans* G-protein-coupled receptor SRA-13 inhibits RAS/MAPK signalling during olfaction and vulval development. Development.

[64] Nakdimon I., Walser M., Fröhli E., Hajnal A. (2012). PTEN negatively regulates MAPK signaling during *Caenorhabditis elegans* vulval development. PLoS Genet..

[65] Hesp K., Smant G., Kammenga J.E. (2015). *Caenorhabditis elegans* DAF-16/FOXO transcription factor and its mammalian homologs associate with age-related disease. Exp. Gerontol..

[66] Sun X., Chen W.D., Wang Y.D. (2017). DAF-16/FOXO Transcription Factor in Aging and Longevity. Front. Pharmacol..

